# Trends in Hepatitis C Virus Infection Prevalence Among People With HIV in Spain Over 2 Decades (2002–2023)

**DOI:** 10.1093/cid/ciaf407

**Published:** 2025-07-24

**Authors:** Juan Berenguer, Chiara Fanciulli, María M Arcos, María J Vivancos, Pere Domingo, Asunción Hernando, Julia Barrado, Pablo Ryan, Jordi Navarro, Rosario Palacios, Luis E Morano, José A Iribarren, Rosa Martínez, María J Galindo, Ignacio de los Santos, Ian López-Cruz, Antonio Rivero, Leire Pérez-Latorre, Livia Giner, María C Fariñas, Coral García, Marta Montero-Alonso, Óscar L Ferrero, Aroa Villoslada, Josefa F Soler-González, José Sanz, Sergio Rodríguez, Juan E Losa, Enrique Bernal, Sergio Veloso, Laura Pérez-Martínez, Fernando Mateos, Laia Arbonés, Raquel Franch, Diana Corps, Cristina Martín, Gerardo Alonso, Marta Clavero-Olmos, Rafael Silvariño, Ramón Teira, Olga Belinchón, Marta de Miguel, Inmaculada Jarrín, Juan González-García

**Affiliations:** Infectious Diseases, Hospital General Universitario Gregorio Marañón, Madrid, Spain; Instituto de Investigación Sanitaria Gregorio Marañón (IiSGM), Madrid, Spain; Centro de Investigación Biomédica en Red de Enfermedades Infecciosas (CIBERINFEC), Madrid, Spain; Infectious Diseases, Hospital General Universitario Gregorio Marañón, Madrid, Spain; Instituto de Investigación Sanitaria Gregorio Marañón (IiSGM), Madrid, Spain; Centro de Investigación Biomédica en Red de Enfermedades Infecciosas (CIBERINFEC), Madrid, Spain; Centro de Investigación Biomédica en Red de Enfermedades Infecciosas (CIBERINFEC), Madrid, Spain; Infectious Diseases, Hospital Universitario La Paz, Madrid, Spain; Instituto de Investigación Sanitaria Hospital La Paz (IdiPAZ), Madrid, Spain; Centro de Investigación Biomédica en Red de Enfermedades Infecciosas (CIBERINFEC), Madrid, Spain; Infectious Diseases, Hospital Universitario Ramón y Cajal, Madrid, Spain; Instituto Ramón y Cajal de Investigación Sanitaria (IRYCIS), Madrid, Spain; Infectious Diseases, Hospital Santa Creu I Sant Pau, Barcelona, Spain; Centro de Investigación Biomédica en Red de Enfermedades Infecciosas (CIBERINFEC), Madrid, Spain; Infectious Diseases, Hospital Universitario 12 de Octubre, Madrid, Spain; Instituto de Investigación Sanitaria Hospital 12 de Octubre (I+12), Madrid, Spain; Centro de Investigación Biomédica en Red de Enfermedades Infecciosas (CIBERINFEC), Madrid, Spain; Infectious Diseases, Hospital Universitario Clínico San Carlos, Madrid, Spain; Instituto de Investigación Sanitaria del Hospital Clínico de San Carlos (IdISSC), Madrid, Spain; Instituto de Investigación Sanitaria Gregorio Marañón (IiSGM), Madrid, Spain; Centro de Investigación Biomédica en Red de Enfermedades Infecciosas (CIBERINFEC), Madrid, Spain; Infectious Diseases, Hospital Universitario Infanta Leonor, Madrid, Spain; Infectious Diseases, Hospital Universitario Vall D’Hebrón, Barcelona, Spain; Infectious Diseases, Hospital Universitario Virgen de la Victoria, Málaga, Spain; Instituto de Investigación Biomédica de Málaga (IBIMA), Málaga, Spain; Infectious Diseases, Hospital Universitario Álvaro Cunqueiro, Vigo, Spain; Instituto de Investigación Sanitaria Galicia Sur (IISGS), Vigo, Spain; Infectious Diseases, Hospital Universitario Donostia, San Sebastián, Spain; Instituto de Investigación Sanitaria Biogipuzkoa, San Sebastián, Spain; Departamento de Medicina, Universidad del País Vasco, San Sebastián, Spain; Infectious Diseases, Hospital Universitario Miguel Servet, Zaragoza, Spain; Infectious Diseases, Hospital Clínico de Valencia, Valencia, Spain; Centro de Investigación Biomédica en Red de Enfermedades Infecciosas (CIBERINFEC), Madrid, Spain; Infectious Diseases, Hospital Universitario de la Princesa, Madrid, Spain; Instituto de Investigación Sanitaria del Hospital de la Princesa (IIS-PRINCESA), Madrid, Spain; Infectious Diseases, Hospital Universitario Doctor Peset, Valencia, Spain; Centro de Investigación Biomédica en Red de Enfermedades Infecciosas (CIBERINFEC), Madrid, Spain; Infectious Diseases, Hospital Universitario Reina Sofía, Córdoba, Spain; Instituto Maimónides de Investigación Biomédica de Córdoba (IMIBIC), Córdoba, Spain; Infectious Diseases, Hospital General Universitario Gregorio Marañón, Madrid, Spain; Instituto de Investigación Sanitaria Gregorio Marañón (IiSGM), Madrid, Spain; Centro de Investigación Biomédica en Red de Enfermedades Infecciosas (CIBERINFEC), Madrid, Spain; Infectious Diseases, Hospital General de Alicante, Alicante, Spain; Centro de Investigación Biomédica en Red de Enfermedades Infecciosas (CIBERINFEC), Madrid, Spain; Infectious Diseases, Hospital Marqués de Valdecilla, Santander, Spain; Infectious Diseases, Hospital Universitario Virgen de las Nieves, Granada, Spain; Infectious Diseases, Hospital Universitario y Politécnico La Fe, Valencia, Spain; Infectious Diseases, Hospital Universitario Basurto, Bilbao, Spain; Infectious Diseases, Hospital Universitario Son Llàtzer, Palma de Mallorca, Spain; Infectious Diseases, Hospital Universitario de Cabueñes, Gijón, Spain; Infectious Diseases, Hospital Universitario Príncipe de Asturias, Alcalá de Henares, Spain; Infectious Diseases, Hospital Universitario de Getafe, Getafe, Spain; Infectious Diseases, Hospital Universitario Fundación Alcorcón, Alcorcón, Spain; Infectious Diseases, Hospital General Universitario Reina Sofía, Murcia, Spain; Infectious Diseases, Hospital Universitario Juan XXIII de Tarragona, Tarragona, Spain; Infectious Diseases, Hospital Universitario San Pedro, Logroño, Spain; Infectious Diseases, Complejo Hospitalario Universitario de Albacete, Albacete, Spain; Infectious Diseases, Hospital de Mataró, Mataró, Spain; Infectious Diseases, Hospital Virgen de la Cinta, Tortosa, Spain; Infectious Diseases, Hospital Universitario de Torrejón, Torrejón de Ardoz, Spain; Infectious Diseases, Hospital Virgen de la Concha, Zamora, Spain; Hospital Rafael Méndez, Lorca, Spain; Hospital Universitario Infanta Elena, Valdemoro, Spain; Hospital San Eloy, Baracaldo, Spain; Hospital Sierrallana, Torrelavega, Spain; Hospital Virgen de la Luz, Cuenca, Spain; Fundación SEIMC/GESIDA, Madrid, Spain; Centro de Investigación Biomédica en Red de Enfermedades Infecciosas (CIBERINFEC), Madrid, Spain; Centro Nacional de Epidemiología—Instituto de Salud Carlos III (ISCIII), Madrid, Spain; Centro de Investigación Biomédica en Red de Enfermedades Infecciosas (CIBERINFEC), Madrid, Spain; Infectious Diseases, Hospital Universitario La Paz, Madrid, Spain; Instituto de Investigación Sanitaria Hospital La Paz (IdiPAZ), Madrid, Spain

**Keywords:** HIV/HCV coinfection, direct-acting antivirals, cirrhosis, injection drug use, men who have sex with men

## Abstract

**Background:**

Hepatitis C virus (HCV) has significantly impacted people with human immunodeficiency virus (HIV). Harm reduction programs, changing transmission patterns, and direct-acting antivirals (DAAs) have profoundly altered HIV/HCV coinfection trends. This study evaluates HCV prevalence among people with HIV in Spain over 2 decades.

**Methods:**

We conducted 9 cross-sectional studies (2002–2023) in 39–43 centers. Sampled individuals were randomly sampled from people with HIV actively followed up at these centers, with proportional allocation. Main outcomes included the prevalence of anti-HCV antibody and active HCV infection (HCV RNA­–positive result).

**Results:**

The reference population ranged from 31 800 to 47 006, with sample sizes of 1260–1867. HIV transmission patterns shifted from 2002 to 2023, with injection drug use decreasing from 55% to 21% and the proportion of men who have sex with men increasing from 17% to 46%. HCV seroprevalence fell from 60.8% to 27.4%, and active infection from 46.3% to 0.9%. In the DAA era (2015–2023), active HCV infection dropped by 100% in heterosexuals, 94% in people who inject drugs, and 71% in men who have sex with men. Treatment uptake increased from 23% in 2002 to 99% by 2023 with all-oral DAAs. The prevalence of cirrhosis among active HCV cases peaked at 23.1% in 2015 but fell to 0% by 2021. Among those achieving sustained virologic response, cirrhosis prevalence was 20.4% in 2023.

**Conclusions:**

HIV/HCV coinfection has drastically declined in Spain, with active HCV infection prevalence <1% since 2021. DAAs were pivotal in this achievement. However, cirrhosis remains a concern among those with sustained virologic response. Ongoing surveillance and prevention efforts are essential to sustain these gains and address residual risks.

## Teaser

Active hepatitis C virus infection in people with human immunodeficiency virus in Spain fell from 54% in 2002 to <1% in 2021–2023. Direct-acting antiviral use since 2015 was pivotal. Ongoing prevention and surveillance are key to maintaining gains.

Hepatitis C virus (HCV) coinfection has been a major health concern for people with human immunodeficiency virus (HIV; PWH) since the onset of the HIV pandemic. Around the turn of the century, seroprevalence levels varied by country, ranging from 30% to >50%, with levels as high as 88% among individuals with a history of injection drug use (IDU) [1, 2]. Although IDU remains the leading route of HCV transmission worldwide [3], high-risk sexual practices among men who have sex with men (MSM), a recognized transmission mechanism since the 1990s [4], account for a growing share of new infections and reinfections among PWH and MSM using HIV preexposure prophylaxis [5, 6].

Direct-acting antivirals (DAAs) have transformed HCV management, particularly among PWH, a group that was historically difficult to treat [7, 8]. Evidence shows that widespread implementation of DAAs is associated with reductions in the prevalence of active HCV infection in this population, suggesting a potential treatment-as-prevention effect [9, 10]. Studies in recent years confirm substantial declines in HCV viremia among PWH in settings with broad DAA access, reflecting meaningful epidemiological changes [11].

Given this evolving epidemiology and changing transmission dynamics among PWH, ongoing surveillance remains essential to inform targeted interventions and support World Health Organization goals for HCV elimination [12]. This study analyzes trends in prevalence of HCV infection among PWH in Spain over 2 decades, using data from 9 studies between 2002 and 2023. The aim is to describe temporal changes in HIV/HCV coinfection and provide insights to guide targeted strategies toward HCV elimination.

## METHODS

This article presents nonpublished data from 2 nationwide HCV prevalence studies among PWH in Spain, conducted in 2021 and 2023, along with a summary of results from 7 similar studies spanning the past 2 decades. The initial 2 studies were conducted in 2002 and 2009, with 5 additional studies since 2015 after national approval of all-oral DAA regimens [13–16].

The primary objective across all studies was to determine the prevalence of anti-HCV antibodies and active HCV infection among PWH as a whole and stratified by the main self-reported HIV transmission groups. Secondary objectives included documenting anti-HCV treatment uptake and characterizing the demographic and clinical features of patients with active HCV infection, as well as the liver disease characteristics among PWH who achieved successful treatment outcomes.

All studies used a standardized cross-sectional design and were conducted in almost the same centers across the country. The target population included PWH actively followed up at participating centers, defined as individuals who had attended ≥1 outpatient visit or experienced hospitalization within 12 months preceding the study. Sample sizes were determined using standard formulas for estimating proportions in cross-sectional surveys [17], informed by prevalence data from previous GeSIDA studies. Parameters included a 95% confidence level, a design effect of 1.0, and target margins of error (ie, absolute precision) ranging from 3.0% in earlier rounds (2002–2009) to 0.5% in the most recent study (2023), as detailed in Table 1. As all studies were conducted within finite populations, finite population correction was systematically applied to initial estimates. From 2016 onward, sample sizes consistently exceeded the minimum required, thereby enhancing statistical precision of prevalence estimates. The sample size at each center was determined using proportional allocation, with sampled individuals selected through simple random sampling.

**Table 1.** Participating Centers, Reference Population, Sample Size and Baseline Characteristics of the Study Population in the 9 Prevalence Studies of Hepatitis C Virus Among People With HIV in Spain

	2002	2009	2015	2016	2017	2018	2019	2021	2023
Participating centers, no.	39	43	41	43	43	43	41	41	39
Reference population, no.	31 800	36 450	35 791	38 904	40 322	40 650	41 973	46 059	47 006
Sample size, no. (%)	1260 (4.0)	1458 (4.0)	1867 (5.2)	1588 (4.1)	1690 (4.2)	1733 (4.3)	1325 (3.2)	1421 (3.1)	1431 (3.0)
Sample size precision, %	3.0	3.0	2.0	2.0	1.5	1.3	1.0	0.8	0.5
Tested for HCV antibodies, %	96.5	99.8	98.7	99.8	99.1	99.3	99.3	98.9	96.2
Male sex assigned at birth, no. (%)	902 (71.6)	1062 (72.8)	1426 (76.4)	1223 (77.0)	1267 (75.0)	1290 (74.4)	999 (75.4)	1065 (75.0)	1125 (78.6)
Age, mean (SD), y	40 (8)	45 (10)	47 (10)	49 (11)	49 (11)	49 (11)	49 (12)	50 (12)	51 (12)
HIV transmission category, no. (%)									
IDU	696 (55.2)	642 (44.0)	573 (30.7)	470 (29.6)	506 (29.9)	495 (28.6)	340 (25.7)	338 (23.8)	299 (20.9)
Heterosexual	311 (24.7)	410 (28.1)	458 (24.5)	376 (23.7)	429 (25.4)	463 (26.7)	338 (25.6)	362 (25.5)	331 (23.1)
MSM	217 (17.2)	351 (24.1)	655 (35.1)	556 (35.0)	578 (34.2)	631 (36.4)	534 (40.3)	609 (42.9)	658 (46.0)
Contaminated blood products	13 (1.0)	7 (0.5)	20 (1.1)	5 (0.3)	6 (0.4)	11 (0.6)	11 (0.8)	6 (0.4)	12 (0.8)
MTCT	0	0	18 (1.0)	11 (0.7)	19 (1.1)	13 (0.8)	19 (1.4)	10 (0.7)	6 (0.4)
Other/unknown	23 (1.8)	48 (3.3)	143 (7.7)	170 (10.7)	152 (9.0)	120 (6.9)	83 (6.3)	96 (6.8)	125 (8.7)
Prior AIDS-defining conditions, no. (%)	416 (33.0)	467 (32.0)	462 (24.7)	402 (25.3)	431 (25.5)	447 (25.8)	268 (20.2)	340 (23.9)	321 (22.4)
ART coverage, no. (%)	1109 (88.0)	1327 (91.0)	1785 (95.6)	1535 (96.7)	1654 (97.9)	1699 (98.0)	1298 (98.0)	1401 (98.6)	1413 (98.7)
HIV-RNA BLQ on ART, no. (%)	580 (46.0)	1035 (71.0)	1625 (87.0)	1415 (89.1)	1510 (88.8)	1580 (91.2)	1202 (90.7)	1332 (93.7)	1320 (92.3)
CD4^+^ T-cell count, median (IQR), cells/mL	444 (276–660)	564	630 (452–852)	670 (470–895)	684 (481–932)	695 (471–915)	694 (481–930)	690 (508–918)	711 (500–939)

Abbreviations: ART, antiretroviral therapy; BLQ, below the limit of quantification; HCV, hepatitis C virus; HIV, human immunodeficiency virus; IDU, injection drug use; IQR, interquartile range; MSM, men who have sex with men; MTCT, mother-to-child transmission.

Data collection methods evolved over time. In 2002 and 2009, data were extracted from medical records using paper case report forms; since 2015, an electronic case report form (REDCap) hosted by Sociedad Española de Enfermedades Infecciosas y Microbiología Clínica (SEIMC)/GeSIDA has been used [18] (Supplementary Appendix 1**)**. Collected variables included demographics, HIV transmission category, CD4^+^ T-cell count, HIV viral load, antiretroviral therapy (ART) status, hepatitis B surface antigen, anti-HCV antibodies, HCV RNA (if applicable), and the most recent blood cell counts and serum biochemical profiles, including aminotransferase levels. Patients receiving HCV treatment at the time of data collection were classified as HCV RNA positive. Additional data for HCV RNA–positive individuals included HCV genotype/subtype, reasons for not receiving treatment (if applicable), and, since 2018, time since diagnosis of active infection (≤1 or >1 year). For those with anti-HCV antibodies but negative HCV RNA results, prior treatment or spontaneous clearance was investigated. Anti-HCV treatment history and outcomes were also documented for those without evidence of spontaneous clearance.

The seroprevalence of HCV was defined as the proportion of PWH who tested positive for anti-HCV antibodies among those with available serological testing. The prevalence of active HCV infection was defined as the proportion of PWH with detectable HCV RNA among those with known HCV antibody status, excluding individuals with a positive antibody result but without HCV RNA testing. Individuals with a negative anti-HCV antibody result were assumed to be HCV RNA negative.

Liver fibrosis was assessed using the fibrosis-4 index and liver stiffness measurements by transient elastography. Cirrhosis was defined by liver biopsy, liver stiffness measurement >12.5 kPa [19], or clinical/biological findings. Fibrosis-4 values ≥3.25 were indicative of advanced liver fibrosis/cirrhosis [20]. In patients with cirrhosis, information was gathered about liver decompensation (ascites, variceal bleeding, and portosystemic encephalopathy), hepatocellular carcinoma (HCC), and liver transplantation.

To assess anti-HCV treatment uptake, we calculated the proportion of patients with current or past HCV infection who received therapy, excluding those with spontaneous clearance. In Spain, interferon-based and all-oral DAA therapies have been hospital based and fully covered by the National Health System. For PWH, treatment has been prescribed and monitored by infectious diseases or internal medicine specialists in hospital HIV clinics. Access to all-oral DAA therapies began in January 2015, initially restricted to patients with severe fibrosis or high transmission risk, and became unrestricted in June 2017.

### Statistical Analysis

A descriptive analysis was performed using frequency tables for categorical variables and mean with SD or median and interquartile range for continuous variables, depending on distribution. Differences in categorical and continuous variables were assessed using χ^2^ tests and *t* or Mann–Whitney tests, respectively. Trends in seropositivity and active HCV infection over time were analyzed using the Cochran-Armitage test. Statistical analyses were performed with Stata software (version 14.0; StataCorp). The study followed STROBE guidelines (Supplementary Table 1).

### Ethical Aspects

The studies were approved by the Ethics Committee for Research of Hospital General Universitario Gregorio Marañón. Informed consent for collecting clinical data was waived, as the study was conducted anonymously and used routine clinical data for scientific publications.

## RESULTS

### Centers, Sampling, and Characteristics of Sampled Individuals

All 9 studies were conducted in 39–43 centers, which remained largely consistent, across 32 cities in 14 of Spain's 17 autonomous communities (Supplementary Appendix 2 and Supplementary Figure 1). Table 1 summarizes key characteristics from the 9 studies, including the number of participating centers, reference populations, sample sizes, sample size precision, and sampled individuals’ characteristics. Overall, the reference population of PWH increased from 31 800 in 2002 to 47 006 in 2023, with sample sizes ranging from 1260 individuals in 2002 to a peak of 1867 in 2015.

The proportion of sampled individuals assigned male sex at birth remained high, ranging from 72% to 79%. The mean age increased from 40 to 51 years over the study period. HIV transmission categories shifted notably: IDU declined from 55.2% to 20.9%, while MSM rose from 17.2% to 46.0% (see also Supplementary Figure 2). ART coverage stayed high, reaching 99.0% in the last 2 years. Among those on ART, HIV-RNA suppression improved from 46.0% to 93.0%, and median CD4^+^ T-cell counts increased from 444/µL to 711/µL.

### Prevalence of Anti-HCV Antibodies and Active HCV Infection Among PWH

**Figure 1.**
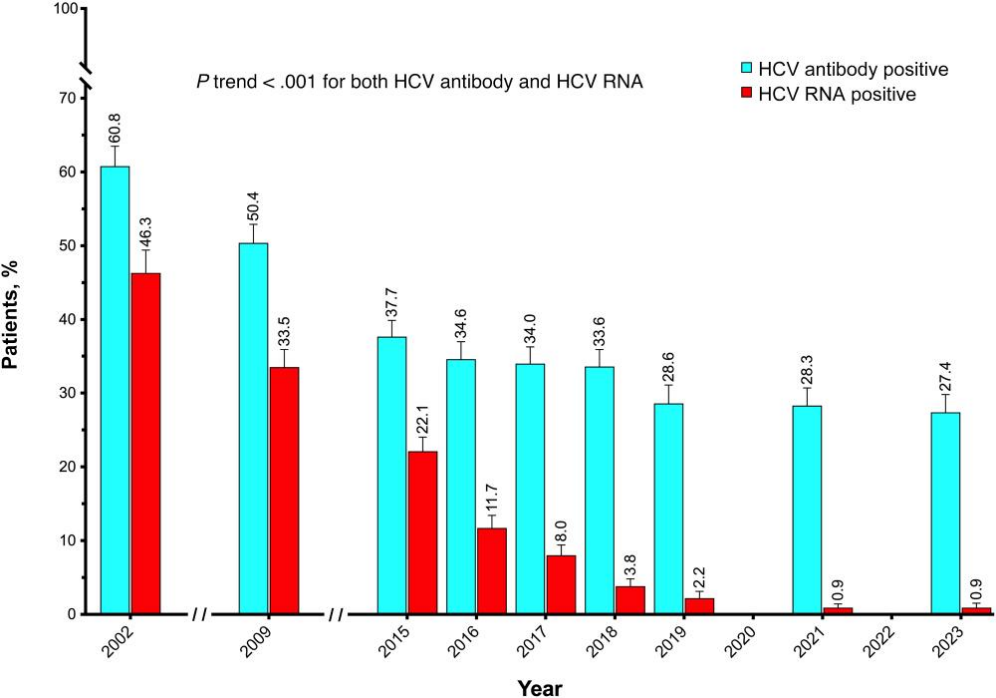
Prevalence of hepatitis C virus (HCV) antibodies and active HCV infection among people with human immunodeficiency virus in Spain, 2002–2023.

The prevalence of anti-HCV antibodies and the prevalence of active HCV infection among PWH in Spain from 2002 to 2023 are shown in Figure 1 and detailed in Supplementary Table 2. In 2002, the overall prevalence of anti-HCV antibodies was 60.8%, compared with 27.4% by 2023. The prevalence of active HCV infection was 46.3% in 2002 and 0.9% from 2021 through 2023.

**Figure 2.**
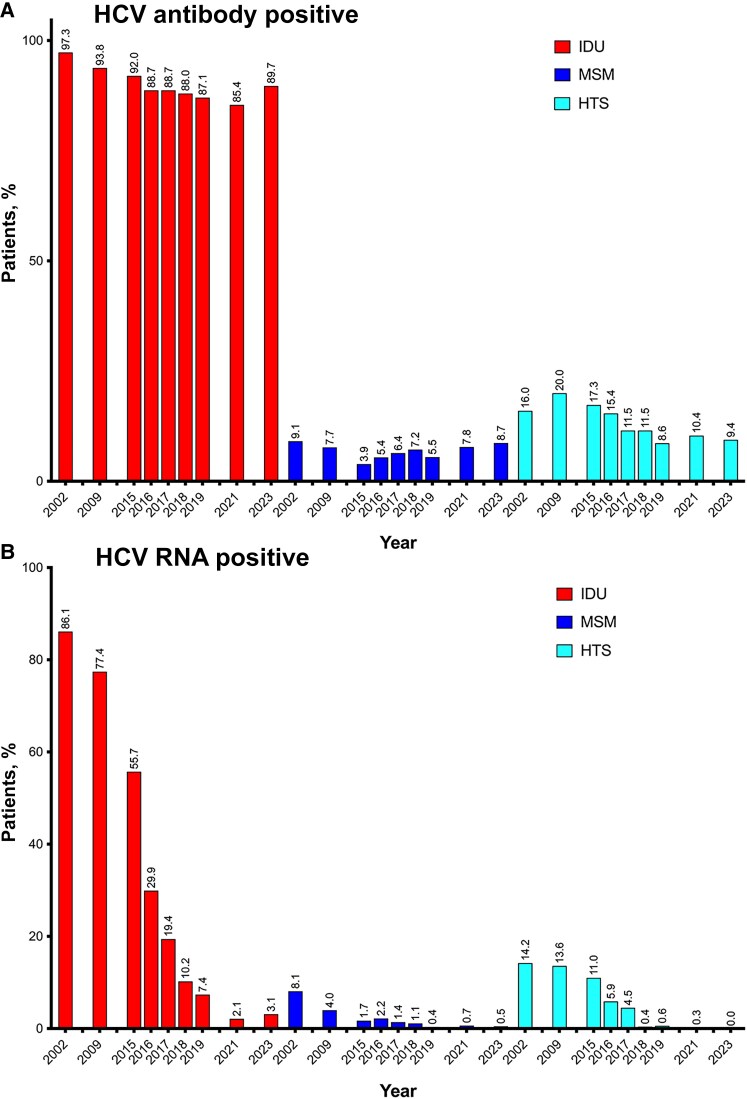
Prevalence of hepatitis C virus (HCV) antibodies and active HCV infection among people with HIV in Spain, 2002–2023, stratified by human immunodeficiency virus transmission categories. Abbreviations: HTS, heterosexual; IDU, injection drug use; MSM, men who have sex with men.

When stratified by HIV transmission category, declines in the prevalence of anti-HCV antibodies were observed over time across all groups (Figure 2*A*). Similarly, the prevalence of active HCV infection decreased in recent years for all transmission categories (Figure 2*B*). Among PWH in the IDU category, the prevalence of active infection was 86.1% in 2002 and 3.1% in 2023. Among those in the heterosexual transmission category, the corresponding figures were 14.2% and 0%, and among MSM, 8.1% and 0.5%. The largest differences were observed during the period when all-oral DAAs became widely available, from 2015 to 2023. During this period, the prevalence of active HCV infection decreased by 94% in the IDU, 100% in the heterosexual, and 71% in the MSM transmission category.

### Anti-HCV Treatment Uptake

**Figure 3.**
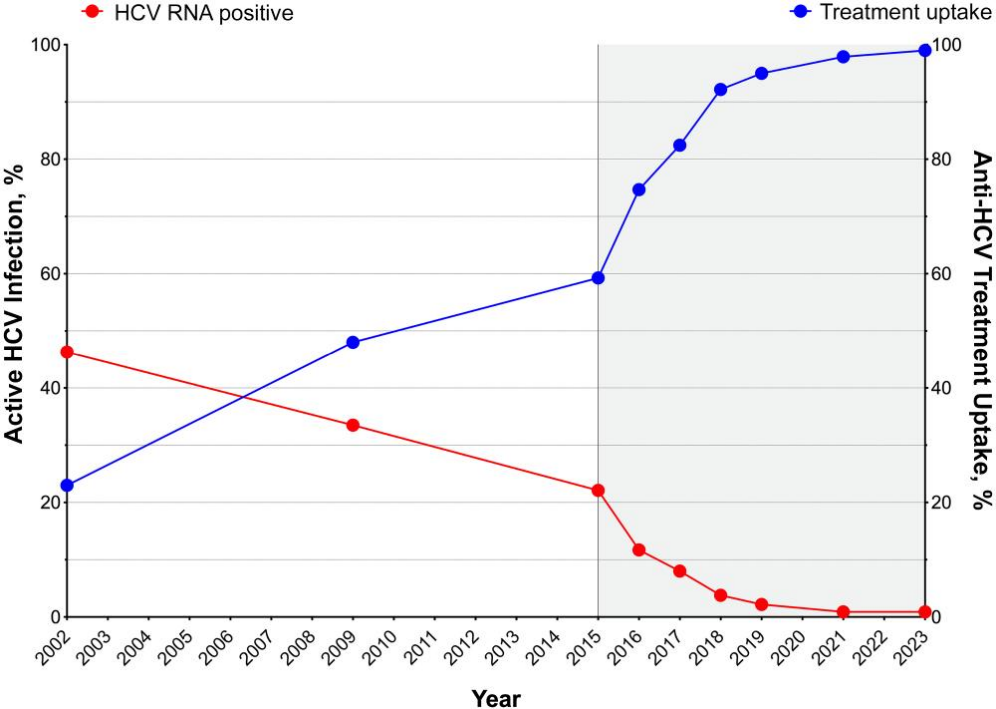
Trends in anti–hepatitis C virus (HCV) treatment uptake (*right y-axis*) and active HCV infection (*left y-axis*) among people with human immunodeficiency virus in Spain from 2002 to 2023. The graph highlights the rapid increase in treatment uptake, particularly after the introduction of direct-acting antivirals in 2015 (*Shaded area extends from 2015 to 2023*), accompanied by a corresponding decline in active HCV infections.

The uptake of anti-HCV treatment has increased steadily and significantly over the years (Figure 3). In 2002, uptake was only 23%, reaching 48% by 2009, when pegylated interferon plus ribavirin was the standard of care for HCV treatment. This upward trend continued, reaching 59% in 2015, following the introduction of combination regimens using pegylated interferon and DAAs, with or without ribavirin, from 2012 onward. After 2015, the rollout of all-oral DAA therapies led to a sharp increase, with treatment uptake peaking at 99% by 2023.

### Demographic and Clinical Characteristics of PWH With Active HCV Infection

Table 2 provides descriptive data on the characteristics of active HCV infections. Active HCV infections with prior sustained virologic response (SVR) were analyzed for the first time in 2015, with 2 cases identified annually in 2015, 2016, and 2017, representing 0.5%, 1.1%, and 1.5% of all active HCV infections, respectively. Four such cases were identified in 2018 (6.1%), while none were reported from 2019 to 2023.

**Table 2.** Characteristics of Active Hepatitis C Virus (HCV) Infections in the 9 Prevalence Studies of HCV Among People With HIV in Spain Conducted Between 2002 and 2023

Characteristic	PWH, No. (%)								
	2002	2009	2015	2016	2017	2018	2019	2021	2023
Active HCV, %^a^	46.3	33.5	22.1	11.7	8.0	3.7	2.2	0.9	0.9
Active HCV infections, no	462	475	402	186	134	66	29	12	12
HIV transmission category									
IDU	418 (90.5)	404 (85.0)	308 (76.6)	140 (75.2)	98 (73.1)	52 (78.8)	25 (86.2)	7 (58.3)	9 (75.0)
MSM	31 (6.7)	12 (2.5)	11 (2.7)	12 (6.5)	8 (6.0)	7 (10.6)	2 (6.9)	4 (33.3)	3 (25.0)
Heterosexual	12 (2.6)	46 (9.8)	49 (12.2)	22 (11.8)	19 (14.2)	2 (3.0)	2 (6.9)	1 (8.3)	0
Other/unknown	1 (0.2)	13 (2.7)	34 (8.5)	12 (6.5)	9 (6.7)	5 (7.6)	0	0	0
With prior SVR	NA	NA	2 (0.5)	2 (1.1)	2 (1.5)	4 (6.1)	0	0	0
Time since diagnosis of active HCV									
≤1 y	NA	NA	NA	NA	NA	3 (4.5)	1 (3.4)	2 (16.7)	6 (50.0)
>1y	NA	NA	NA	NA	NA	62 (93.9)	24 (82.8)	9 (75.0)	4 (33.3)
Unknown	NA	NA	NA	NA	NA	1 (1.5)	4 (13.8)	1 (8.3)	2 (16.7)
HCV genotype									
Unknown	95 (20.6)	27 (5.7)	35 (8.7)	10 (5.4)	14 (10.4)	7 (10.6)	5 (17.2)	4 (33.3)	2 (16.7)
Known	367 (79.4)	448 (94.3)	367 (91.3)	176 (94.6)	120 (89.6)	59 (89.4)	24 (82.8)	8 (66.7)	10 (83.3)
1a	122 (33.2)	155 (34.6)	143 (39.0)	82 (46.6)	49 (40.8)	22 (37.3)	8 (33.3)	3 (37.5)	4 (40.0)
4	66 (18.0)	90 (20.1)	90 (24.5)	39 (22.2)	28 (23.3)	16 (27.1)	7 (29.2)	1 (12.5)	3 (30.0)
1b	61 (16.6)	75 (16.7)	69 (18.8)	24 (13.6)	12 (10.0)	11 (19.6)	4 (16.7)	1 (12.5)	2 (20.0)
3	99 (17.0)	104 (23.2)	57 (15.5)	28 (15.9)	26 (21.7)	9 (15.2)	3 (12.5)	1 (12.5)	0
2	7 (1.9)	6 (1.3)	5 (1.4)	3 (1.7)	3 (2.5)	1 (1.7)	0	0	1 (10.0)
Mixed	12 (3.3)	18 (4.0)	3 (0.8)	0	2 (1.7)	0	2 (8.3)	2 (25.0)	0
Cirrhosis^b^	42 (9.1)	91 (19.2)	93 (23.1)	28 (15.0)	14 (10.4)	8 (12.1)	3 (10.3)	0	0
Decompensated cirrhosis	14 (3.0)	20 (4.2)	17 (4.2)	4 (2.2)	4 (3.0)	2 (3.0)	0	0	0
Cirrhosis with HCC	1 (0.2)	5 (1.1)	3 (0.7)	1 (0.5)	1 (0.7)	1 (1.5)	0	0	0

Abbreviations: HCC, hepatocellular carcinoma; HCV, hepatitis C virus; HIV, human immunodeficiency virus; MSM, men who have sex with men; NA, not available; PWH, people with HIV; SVR, sustained viral response (after anti-HCV therapy).

^a^The proportion of PWH with detectable HCV RNA among those with known HCV antibody status, excluding individuals with a positive antibody result but without HCV RNA testing. Individuals with a negative anti-HCV antibody result were assumed to be HCV RNA negative.

^b^Diagnostic methods evolved from liver biopsy and clinical criteria in 2002 to the inclusion of liver stiffness measurement in later years.

Analysis of the time since diagnosis among active HCV infections, available from 2018 onward, reveals an increasing proportion of recently diagnosed infections (≤1 year) in later study rounds: 3 cases (4.5%) in 2018, 1 (3.4%) in 2019, 2 (16.7%) in 2021, and 6 (50.0%) in 2023.

Between 2015 and 2023, the proportion of PWH with active HCV infection receiving anti-HCV therapy at the time of data collection ranged from 17% to 58%, varying by year. From 2018 onward, when reasons for nontreatment were systematically collected, the most common was pending initiation (29%–50% annually). Other frequent reasons included patient refusal, loss to follow-up, and medical decision (see Supplementary Table 3 for details).

Genotype 1a remained the most frequent genotype throughout the study period, ranging from 33.2% in 2002 to 40.0% in 2023. Genotype 4 was consistently the second most common in all years except 2009, with proportions varying between 18.0% in 2002 and 30.0% in 2023. The third most frequent genotype alternated between 1b and 3, while genotype 2 was consistently infrequent.

Among patients with active HCV infection, the proportion with a diagnosis of liver cirrhosis was 9.1% in 2002 and 23.1% in 2015, with no cases reported from 2021 to 2023. Diagnostic practices changed over time, evolving from liver biopsy and clinical criteria in 2002 to the inclusion of liver stiffness measurements in subsequent years. The proportion of patients with decompensated cirrhosis was 4.2% in 2015 and 0% from 2019 onward. Similarly, cirrhosis with HCC was reported in 0.2% in 2002 and 1.5% in 2018, with no cases identified since 2019.

### Liver Disease Characteristics Among PWH With Successful Treatment Outcomes

The proportion of patients with a history of prior HCV infection and SVR following anti-HCV therapy, which remained <5% in the 2 surveys conducted before the introduction of all-oral DAAs, increased after 2015 and exceeded 20% in all study rounds from 2017 onward (Table 3 and Supplementary Figure 3). Data on liver disease among these patients, available since 2015, indicate that the proportion diagnosed with cirrhosis was 31.5% in 2016 and 20.4% in 2023. The proportion of patients with decompensated cirrhosis was 1.2% in 2015 and 4.7% in 2023 The proportion of patients with HCC among those with SVR remained generally <1%, except in 2023, when it was 2.3%.

**Table 3.** **Characteristics of Liver Disease Among People With HIV and a Documented History of Sustained Virologic Response After Anti–Hepatitis C Virus** (**HCV) Therapy: Results From 9 National Cross-Sectional HCV Studies in Spain, 2002–2023**

Characteristic	PWH, No. (%)^a^								
	2002 (n = 1260)	2005 (n = 1458)	2015 (n = 1867)	2016 (n = 1588)	2017 (n = 1690)	2018 (n = 1733)	2019 (n = 1325)	2021 (n = 1421)	2023 (n = 1431)
Prior HCV infection and SVR	8 (0.6)	61 (4.2)	170 (9.1)	292 (18.4)	344 (20.4)	406 (23.4)	291 (22.0)	320 (22.5)	299 (20.9)
Liver complications in PWH with prior SVR									
Cirrhosis	NA	NA	39 (22.9)	92 (31.5)	97 (28.2)	107 (26.4)	68 (23.4)	59 (18.4)	61 (20.4)
Decompensated cirrhosis	NA	NA	2 (1.2)	8 (2.7)	8 (2.3)	14 (3.4)	11 (3.8)	6 (1.9)	14 (4.7)
Cirrhosis with HCC	NA	NA	1 (0.6)	1 (0.3)	2 (0.6)	3 (0.7)	6 (2.1)	1 (0.3)	7 (2.3)

Abbreviations: HCC, hepatocellular carcinoma; HCV, hepatitis C virus; NA, not available; PWH, people with human immunodeficiency virus; SVR, sustained virologic response (after anti-HCV therapy).

^a^Percentages for the first row are calculated using the total number of sampled PWH as the denominator; percentages in subsequent rows use the number of PWH with documented SVR as the denominator.

## DISCUSSION

This study describes HCV antibody seroprevalence and active HCV infection prevalence among PWH in Spain based on 9 cross-sectional surveys over 2 decades. The proportion of individuals with active HCV infection was 46.3% in 2002 and 0.9% in 2021 and 2023, paralleling an increase in treatment uptake from 23% to 99% during the same period. The largest difference in active infection proportions occurred between 2015 and 2023, coinciding with the widespread rollout of all-oral DAA therapies. This pattern is consistent with international data linking high DAA coverage to reduced HCV prevalence and incidence [10, 21–25].

Although comparable trends in the general Spanish population are lacking, a 2017–2018 estimate placed active HCV prevalence at 0.22%, early in the DAA era [26]. While not directly comparable, national data also show post-DAA declines in HCV-related hospitalizations [27, 28], liver transplantations [29], and liver-related deaths [30].

The number of active HCV infections attributable to the IDU transmission category among PWH declined markedly over the study period. Although this group historically accounted for most active infections, the absolute number of active infections in this category has substantially decreased in recent years. The decline in HCV seroprevalence and active infections over the past 2 decades likely reflects the combined influence of several factors. In earlier years, high mortality among PWH with HCV acquired through IDU played a major role [31]. Concurrently, harm reduction programs were associated with a sharp decline in HIV diagnoses linked to IDU, while new HIV infections increasingly occurred among MSM, a group with lower HCV coinfection prevalence [32]. Since 2015, the widespread use of DAAs has likely further reduced active infections. Nonetheless, the proportion of active HCV remains high among individuals with a history of IDU, indicating ongoing vulnerability.

Among individuals in the heterosexual transmission category, the proportion with active HCV infection remained higher than that observed among MSM until 2017. However, since 2018, the prevalence of active HCV infection in the heterosexual remained <1%, reaching 0% in 2023. Although sexual HCV transmission to heterosexual partners in stable relationships is rare [33], cofactors such as HIV or other sexually transmitted infections, blood-exposing sexual practices, partner IDU, and high partner turnover may increase risk, especially in high-prevalence settings [34–38]. While sexual transmission was not confirmed in these studies, molecular evidence supports a case of male-to-female transmission following oral, vaginal, and anal intercourse [39]. Misclassification may occur, as some self-identified heterosexual men engage in MSM networks [40] or underreport IDU [41]. Moreover, up to 1 in 5 MSM with HIV report female partners in the past year [42], suggesting that female partners of MSM could be exposed to HCV.

In contrast, the MSM transmission category, historically contributing less to overall HIV/HCV coinfection burden in Spain showed smaller differences in active HCV proportions between 2015 and 2023 compared with other transmission categories. As a result, MSM currently represent a larger share of PWH with active HCV infection, suggesting a shift in the distribution of HIV/HCV coinfection. These observations may reflect gaps in prevention and treatment uptake among individuals within this transmission category who engage in high-risk behaviors for HCV transmission, including condomless receptive anal intercourse, receptive fisting, group sex, and the use of nonprescription drugs in a sexual context [43, 44].

The characteristics of active HCV infections varied across study rounds. In earlier years, most infections occurred in the IDU transmission category and were often associated with advanced liver fibrosis. In 2015, nearly 25% of active infections were linked to cirrhosis, including cases with decompensation or HCC. No cirrhosis was recorded among individuals with active HCV infection in 2021 and 2023. Since 2018, a higher proportion of active infections have been recently diagnosed, particularly among MSM. This pattern may reflect ongoing transmission or improved surveillance, although small number of cases and the lack of longitudinal data limit interpretation.

The proportion of active HCV infections with prior SVR may represent reinfections. The higher relative proportion up to 2018, despite fewer overall infections, suggests ongoing risk or gaps in prevention. The absence of such cases from 2019 to 2023 may reflect improved prevention, behavioral changes, or treatment-as-prevention effects, although underdetection cannot be excluded. Notably, data from Madrid—the region with the largest PWH population in Spain—showed substantially higher risk of reinfection among MSM than among individuals with an IDU history during the early DAA era [45].

Although DAA scale-up has substantially reduced the number of active infections, the burden of liver disease remains relevant. Many individuals with SVR still have cirrhosis, placing them at continued risk for HCC and liver decompensation, particularly in the presence of cofactors such as alcohol use, metabolic dysfunction–associated liver disease, and drug-induced liver injury [46]. As the population of PWH ages, addressing residual liver disease will require long-term monitoring and integrated care models.

This study has several limitations. Direct assessment of HCV incidence was not feasible, as it requires a prospective cohort design. Nonetheless, repeated cross-sectional studies within a defined population offer a valuable means of estimating incidence and monitoring disease burden over time [47]. The design also precluded detailed assessment of reinfections, which we inferred by identifying active HCV infections in individuals with prior SVR. Classification by HIV transmission relied on self-reported data, which may not reflect the actual route of HCV acquisition and could limit interpretation of transmission-specific comparisons. Mortality could not be accounted for and may have influenced the composition of the study population over time, particularly among individuals with IDU history or advanced liver disease. Despite these limitations, the study's strengths include the use of consistent methods across study periods and a large, representative sample of PWH from diverse regions in Spain. Samples, randomly selected each year from reference populations representing approximately one-third of PWH in Spain during the same year, ensure broad generalizability of the findings.

In conclusion, cross-sectional data over the past 2 decades show a marked decline in HCV seroprevalence and active infections among PWH in Spain. This pattern likely reflects shifts in HIV transmission patterns, high mortality rates among those with a history of IDU, and the widespread use of DAAs. Despite these improvements, ongoing transmission, particularly among MSM, and the burden of cirrhosis after cure continue to pose challenges. Continued surveillance, prevention, and accessible care remain essential to sustain progress.

## Supplementary Material

ciaf407_Supplementary_Data

## Supplementary Data

Supplementary materials are available at *Clinical Infectious Diseases* online. Consisting of data provided by the authors to benefit the reader, the posted materials are not copyedited and are the sole responsibility of the authors, so questions or comments should be addressed to the corresponding author.

## Notes

***Acknowledgments***. The authors thank all members of the GeSIDA 8514 Study Group for their invaluable contributions (Supplementary Appendix 2). They also extend their gratitude to the healthcare professionals caring for people with human immunodeficiency virus HIV at the participating centers.

***Author contributions***. Study conception and design: J. Berenguer and J. G. G. Acquisition of data: C. F., M. M. Arcos, M. J. V., P. D., A. H., J. Barrado, P. R., J. N., R. P., L. E. M., J. A. I., R. M., M. J. G., I. d. l. S., I. L. C., A. R., L. P. L., L. G., M. C. F., C. G., M. Montero-Alonso, O. L. F., A. V., J. F. S. G., J. S., S. R., J. E. L., E. B., S. V., L. P. M., F. M., L. A., R. F., D. C., C. M., G. A., M. C. O., R. S., R. T., and O. B. Database construction in REDCap, quality control, and query resolution: M. d. M. Full access to all study data, data analysis and interpretation, and verification of the data's accuracy: J. Berenguer, I. J., and J. G. G. Drafting of the manuscript: J. Berenguer. Funding acquisition: J. Berenguer and J. G. G. Critical revision of the manuscript for intellectual content and approval of the final manuscript for submission: All authors.

***Data availability***. Data collected for this study from 2015 onward, including deidentified sampled individuals’ data and a data dictionary defining each field, can be made available to others on reasonable request. Additional related documents, such as the study protocol and statistical analysis plan, will also be provided. These data will be available beginning with the publication of this manuscript. Requests for access can be directed to hesteban@f-sg.org. Data sharing will be subject to approval of a research proposal, a signed data access agreement, and compliance with ethical guidelines to ensure sampled individuals’ confidentiality and data protection.

***Disclaimer.*** The funders had no role in the design, conduct, or analysis of the study, nor in the decision to submit the manuscript for publication.

***Financial support***. This work was supported by Fundación para la Investigación y la Prevención del Sida en España (grants 12185/01 and 12689/07), Instituto de Salud Carlos III (grants RD16/0025/0017 and RD16/0025/0018), and the GILEAD Fellowship Programme (Spain) (grant GLD14-00279).
