# Supplementary material for: Trends in Hepatitis C Virus Infection Prevalence Among People With HIV in Spain Over 2 Decades (2002–2023)
Source: Clin Infect Dis. 2025 Jul 24;82(3):e511–20. doi: 10.1093/cid/ciaf407 (PMC13017309; doi:10.1093/cid/ciaf407)
Supplement: ciaf407_Supplementary_Data [file ciaf407_supplementary_data.pdf]

Table of contents

Item	Pages
**Table S1**. STROBE Statement—Checklist of items that should be included in reports of cohort studies	**2**
**Table S2**. Prevalence of HCV antibodies and active HCV infection among parcipants in the nine prevalence studies of HCV among PWH in Spain conducted between 2002 and 2023	**3**
**Table S3**. Annual number of PWH with active HCV infection, treatment status, and reasons for not receiving antiviral therapy (2015 – 2023)	**4**
**Figure S1.** Map showing the locations of 43 medical centers that consistently participated in nine HIV/HCV prevalence studies conducted across different Spanish autonomous communities between 2002 and 2023.	**5**
**Figure S2.** Distribution of HIV transmission categories among participants in nine HCV prevalence studies conducted among people with HIV in Spain from 2002 to 2023.	**6**
**Figure S3.** Liver disease characteristics among PWH with successful treatment outcomes	**7**
**Appendix 1**. Electronic Case Report Form (REDCap®), hosted by SEIMC/GeSIDA, used for data collection in this study at participating centers (in Spanish).	**8-12**
**Appendix 2.** Participating centers in the nine prevalence studies of HCV in Spain conducted between 2002 and 2023.	**13**
**Appendix 2.** GeSIDA 8514 Study Group.	**14**

Table S1. STROBE Statement—Checklist of items that should be included in reports of cohort studies

	Item	Recommendation	Manuscript location
**Title and abstract**	1	(*a*) Indicate the study's design with a commonly used term in the title or the abstract	Abstract
		(*b*) Provide in the abstract an informative and balanced summary of what was done and what was found	Abstract
**Introduction**			
Background/rationale	2	Explain the scientific background and rationale for the investigation being reported	Introduction
Objectives	3	State specific objectives, including any prespecified hypotheses	Introduction
**Methods**			
Study design	4	Present key elements of study design early in the paper	Methods
Setting	5	Describe the setting, locations, and relevant dates, including periods of recruitment, exposure, follow-up, and data collection	Methods and Supplementary Material
Participants	6	(*a*) Give the eligibility criteria, and the sources and methods of selection of participants. Describe methods of follow-up	Methods
		(*b*) For matched studies, give matching criteria and number of exposed and unexposed	Non applicable
Variables	7	Clearly define all outcomes, exposures, predictors, potential confounders, and effect modifiers. Give diagnostic criteria, if applicable	Methods
Data sources/ measurement	8*	For each variable of interest, give sources of data and details of methods of assessment (measurement). Describe comparability of assessment methods if there is more than one group	Methods
Bias	9	Describe any efforts to address potential sources of bias	Non applicable
Study size	10	Explain how the study size was arrived at	Non applicable
Quantitative variables	11	Explain how quantitative variables were handled in the analyses. If applicable, describe which groupings were chosen and why	Methods (Statistical analysis)
Statistical methods	12	(*a*) Describe all statistical methods, including those used to control for confounding	Methods (Statistical analysis)
		(*b*) Describe any methods used to examine subgroups and interactions	Non applicable
		(*c*) Explain how missing data were addressed	Non applicable
		(*d*) If applicable, explain how loss to follow-up was addressed	Non applicable
		(*e*) Describe any sensitivity analyses	Non applicable
**Results**			
Participants	13*	(a) Report numbers of individuals at each stage of study—e.g. numbers potentially eligible, examined for eligibility, confirmed eligible, included in the study, completing follow-up, and analyzed	Results (Centers, sampling, and participant characteristics)
		(b) Give reasons for non-participation at each stage	Non applicable
		(c) Consider use of a flow diagram	Non applicable
Descriptive data	14*	(a) Give characteristics of study participants (e.g. demographic, clinical, social) and information on exposures and potential confounders	Results (Centers, sampling, and participant characteristics), Table 1. Supplementary Table S3.
		(b) Indicate number of participants with missing data for each variable of interest	Non applicable
		(c) Summarize follow-up time (e.g., average and total amount)	Non applicable
Outcome data	15*	Report numbers of outcome events or summary measures over time	Results (Prevalence of HIV/HCV coinfection), Figure 1, Supplementary Table S2.
Main results	16	(*a*) Give unadjusted estimates and, if applicable, confounder-adjusted estimates and their precision (eg, 95% confidence interval). Make clear which confounders were adjusted for and why they were included	Results (Prevalence of HIV/HCV coinfection), Figure 1, Supplementary Table S2.
		(*b*) Report category boundaries when continuous variables were categorized	Non applicable
		(*c*) If relevant, consider translating estimates of relative risk into absolute risk for a meaningful time period	Non applicable
Other analyses	17	Report other analyses done—e.g. analyses of subgroups and interactions, and sensitivity analyses	Results (Prevalence of HIV/HCV coinfection, Access to anti-HCV treatment, Characteristics of active HCV infections, Residual burden of liver disease after SVR), Figure 2, Figure 3, Table 2, Table 3
**Discussion**			
Key results	18	Summarize key results with reference to study objectives	Discussion
Limitations	19	Discuss limitations of the study, taking into account sources of potential bias or imprecision. Discuss both direction and magnitude of any potential bias	Discussion
Interpretation	20	Give a cautious overall interpretation of results considering objectives, limitations, multiplicity of analyses, results from similar studies, and other relevant evidence	Discussion
Generalizability	21	Discuss the generalizability (external validity) of the study results	Discussion
**Other information**			
Funding	22	Give the source of funding and the role of the funders for the present study and, if applicable, for the original study on which the present article is based	Acknowledgements

Note: An Explanation and Elaboration article discusses each checklist item and gives methodological background and published examples of transparent reporting. The STROBE checklist is best used in conjunction with this article (freely available on the Web sites of PLoS Medicine at http://www.plosmedicine.org/, Annals of Internal Medicine at http://www.annals.org/, and Epidemiology at http://www.epidem.com/). Information on the STROBE Initiative is available at http://www.strobe-statement.org

Table S2. Prevalence of HCV antibodies and active HCV infection among sampled individuals in nine cross-sectional studies of HCV in PWH in Spain, 2002–2023

Table S2a. Sample size and results of HCV antibody and RNA testing by study year

year	Sample size	Nº with HCV Ab unknown	Nº with HCV Ab known	Nº HCV Ab negative	Nº HCV Ab positive	Nº HCV -RNA known	Nº HCV-RNA unknown	N º HCV RNA positive	Nº HCV RNA negative post SVR	Nº HCV RNA negative spontaneous clearance
**2002**	1260	44	1216	477	739	520	219	462	8	50
**2009**	1458	3	1455	722	733	698	35	475	61	162
**2015**	1867	24	1843	1148	695	674	21	402	170	102
**2016**	1588	3	1585	1037	548	546	2	186	292	68
**2017**	1690	15	1675	1106	569	569	0	134	344	91
**2018**	1733	12	1721	1143	578	577	1	66	407	104
**2019**	1325	9	1316	940	376	375	1	29	291	55
**2021**	1421	15	1406	1008	398	397	1	12	320	65
**2023**	1431	55	1376	999	377	374	3	12	299	63

*In 2002 and 2009 no differentiation was made between those with HCV RNA negative result due to SVR or spontaneous clearance.

Table S2b. Prevalence of HCV antibodies among PWH (HCV seroprevalence)

year	Numerator: Nº HCV Ab positive	Denominator: Nº with HCV Ab known	Prevalence	95% CI
**2002**	739	1216	60.8	58.0 – 63.5
**2009**	733	1455	50.4	47.8 – 52.9
**2015**	695	1843	37.7	35.5 - 39.9
**2016**	548	1585	34.6	32.3 – 37.0
**2017**	569	1675	34.0	31.7 - 36.3
**2018**	578	1721	33.6	31.4 - 35.9
**2019**	376	1316	28.6	26.2 - 31.1
**2021**	398	1406	28.3	26.0 - 30.7
**2023**	377	1376	27.4	25.1 - 29.8

Table S2c. Prevalence of active HCV infection among PWH (HCV RNA positivity)

year	Numerator: N º HCV RNA positive	Denominator: Nº with HCV Ab known excluding Nº HCV-RNA unknown	Prevalence	95% CI
**2002**	462	997	46.3	43.3 – 49.4
**2009**	475	1420	33.5	31.0 – 35.9
**2015**	402	1822	22.1	20.2 - 24.0
**2016**	186	1583	11.7	10.2 - 13.4
**2017**	134	1675	8.0	6.8 - 9.4
**2018**	66	1720	3.8	3.0 - 4.8
**2019**	29	1315	2.2	1.5 - 3.1
**2021**	12	1405	0.9	0.5 - 1.4
**2023**	12	1373	0.9	0.5 - 1.5

Notes:

1. The seroprevalence of HCV was defined as the proportion of PWH who tested positive for anti-HCV antibodies among those with available serological testing.
2. The prevalence of active HCV infection was defined as the proportion of PWH with detectable HCV RNA among those with known HCV antibody status, excluding individuals with a positive antibody result but without HCV RNA testing. Individuals with a negative anti-HCV antibody result were assumed to be HCV RNA negative
3. Proportions were calculated with 95% confidence intervals using the Wilson method for HCV antibodies and the Jeffreys method for HCV RNA, selected for their accuracy in moderate and low prevalence settings, respectively.

Table S3. Annual number of PWH with active HCV infection, anti-HCV treatment status at the time of data collection, and reasons for not receiving anti-HCV treatment (2015 – 2023)

Year	PWH with active HCV infection	Receiving anti HCV treatment, n (%)*	Not receiving anti HCV treatment, n (%)	Reasons for not receiving anti HCV treatment, n (% of those not treated)
**2015**	502	98 (19.5)	404 (80.5)	Not available
**2016**	186	41 (22.0)	145 (78.0)	Not available
**2017**	134	36 (26.9)	98 (73.1)	Not available
**2018**	66	23 (34.8%)	43 (65.2%)	Pending initiation: 19 (44.2%)<br>Patient refusal: 12 (27.9%)<br>Loss to follow-up: 7 (16.3%)<br>Medical decision: 5 (11.6%)
**2019**	29	11 (37.9%)	18 (62.1%)	Pending initiation: 4 (22.2%)<br>Patient refusal: 2 (11.1%)<br>Loss to follow-up: 3 (16.7%)<br>Medical decision: 5 (27.8%)<br>Other: 4 (22.2%)
**2021**	12	2 (16.7%)	10 (83.3%)	Pending initiation: 3 (33.3%)<br>Patient refusal: 1 (11.1%)<br>Loss to follow-up: 1 (11.1%)<br>Medical decision: 1 (11.1%)<br>Unknown: 3 (33.3%)
**2023**	12	7 (58.3%)	5 (41.7%)	Pending initiation: 2 (50.0%)<br>Patient refusal: 1 (25.0%)<br>Other: 1 (25.0%)<br>Unknown: 1

*By definition, patients undergoing HCV treatment at the time of data collection were classified as HCV-RNA positive.

Figure S1. Map showing the locations of 43 medical centers that consistently participated in nine HIV/HCV prevalence studies conducted across different Spanish autonomous communities between 2002 and 2023.

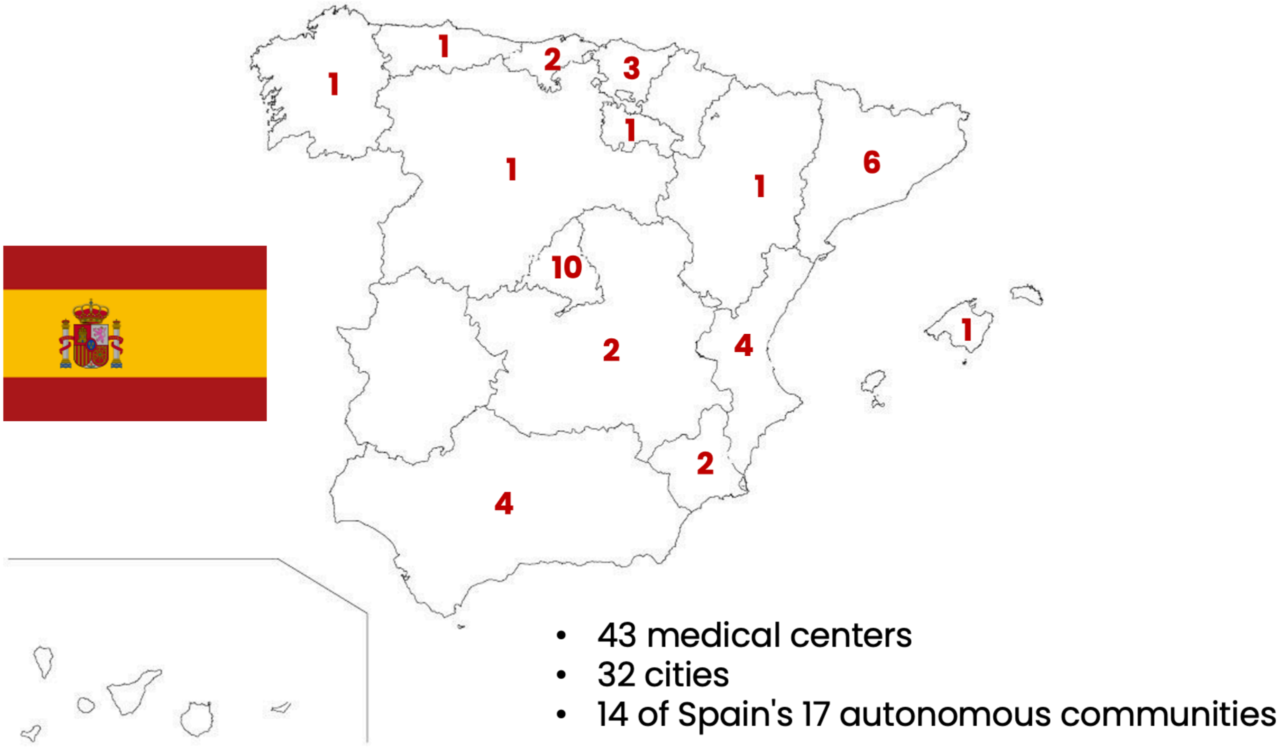

Figure S2. Distribution of HIV transmission categories among participants in nine HCV prevalence studies conducted among people with HIV in Spain from 2002 to 2023.

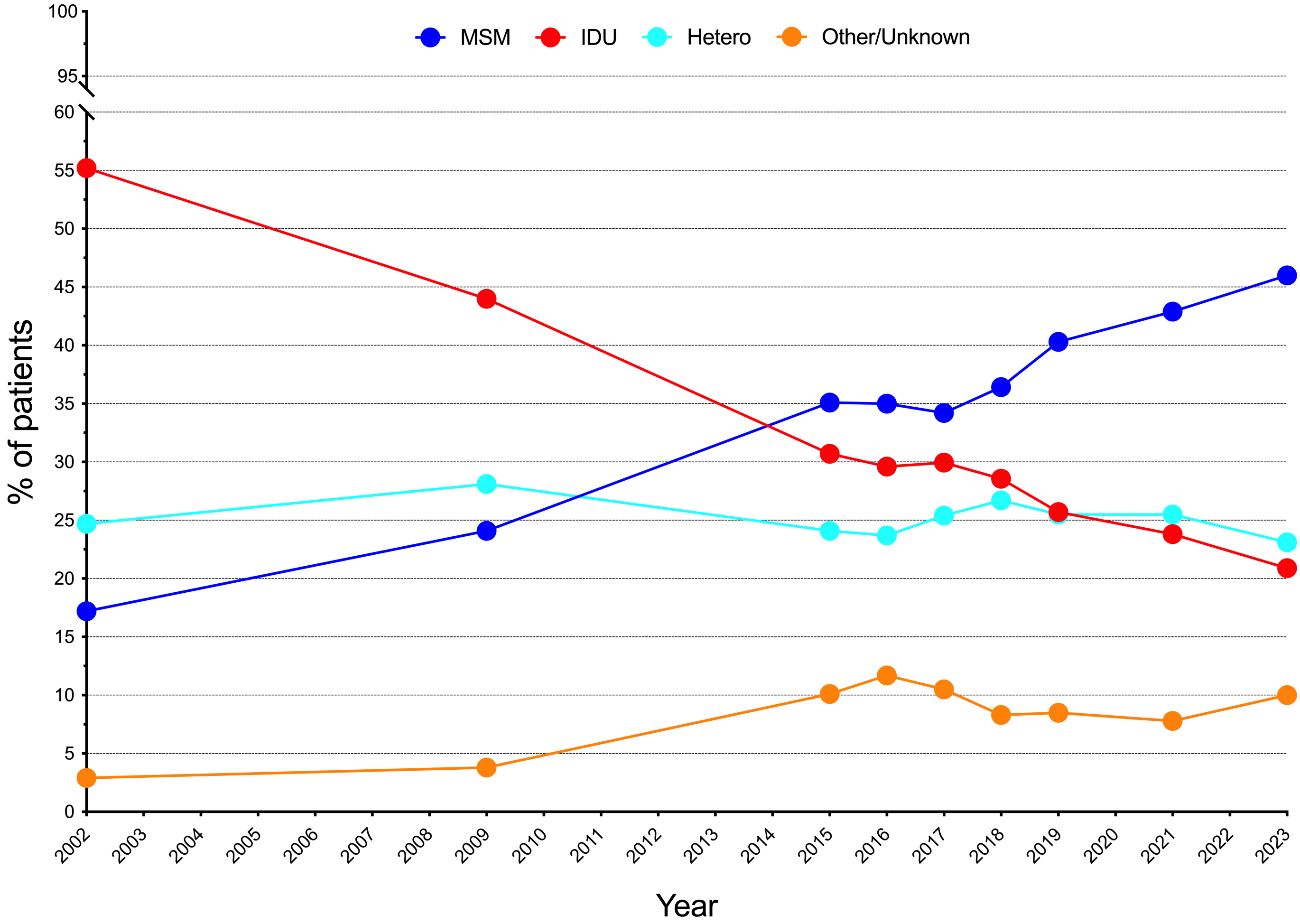

Figure S3. Liver disease characteristics among PWH with successful treatment outcomes

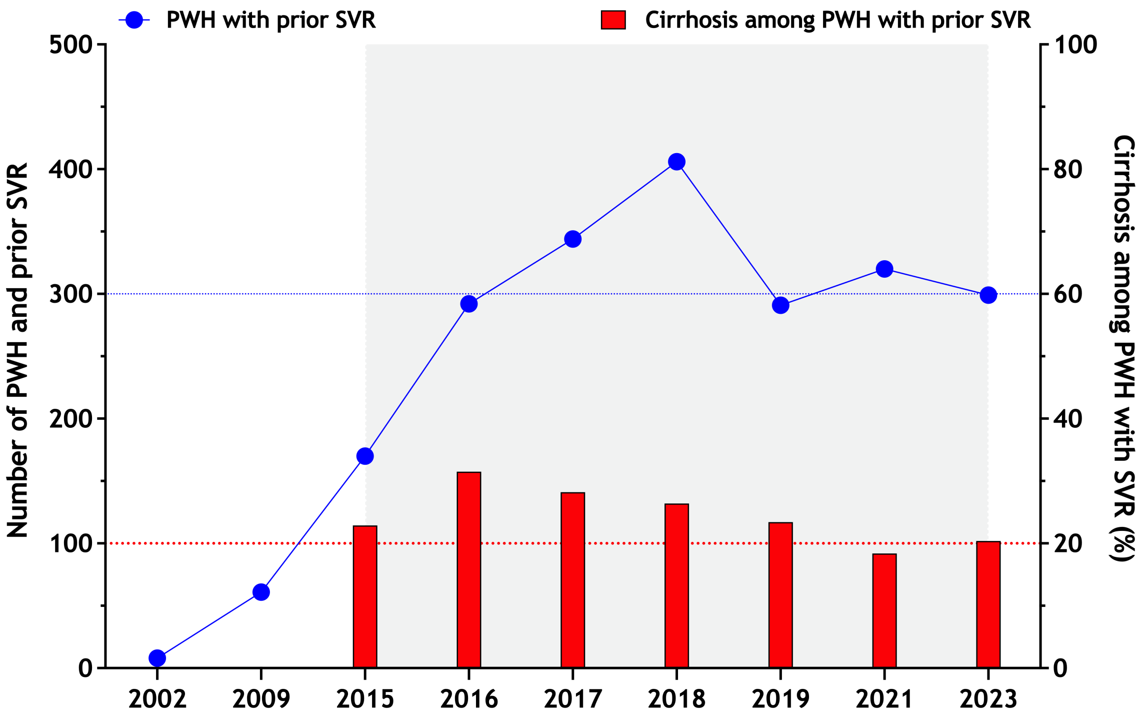

	2002	2009	2015	2016	2017	2018	2019	2021	2023
**PWH sampled**	1260	1458	1867	1588	1690	1733	1325	1421	1431
**PWH with prior SVR**	8	61	170	292	344	407	291	320	299
**PWH with prior SVR & Cirrhosis**	NA	NA	39	92	97	107	68	59	61

Note: This figure summarizes data on cirrhosis in patients with prior HCV infection and SVR after anti-HCV therapy. The blue line, plotted against the left y-axis, shows the number of participants with prior HCV infection and SVR, while the red bars, plotted against the right y-axis, indicate the percentage with cirrhosis. Data available since 2015 show that cirrhosis prevalence remained substantial, peaking at 31.5% in 2016 before gradually declining and stabilizing at 20% in 2021 and 2023.

Appendix 1. Electronic Case Report Form (REDCap®), hosted by SEIMC/GeSIDA, used for data collection in this study at participating centers (in Spanish).

GESIDA 8514. PREVALENCIA VIH/VHC. Año 2023
Page 1

REGISTRO DE PACIENTE

Record ID	__________________________________

Por definición en este estudio:

1) Todo paciente en tratamiento anti-VHC actual o pendiente de resultado de RVS12 se considera infección activa (ARN-VHC+)

2) Un valor de fibroscan > 12,5 kPa es diagnóstico de cirrosis hepática

Fecha de nacimiento:	__________________________________
Sexo:	◯ Hombre ◯ Mujer
Vía de transmisión VIH:	◯ Heterosexual<br>◯ Homo-Bisexual<br>◯ ADVP<br>◯ Vertical<br>◯ Transfusiones<br>◯ Otros / Desconocido
Enfermedades C del CDC (previa o actual):	◯ Yes ◯ No
CD4/mm3 actual:	__________________________________<br>(Última determinación realizada durante el último año)
ARN VIH actual:	◯ < 50 copias/mL<br>◯ 50 - 200 copias/mL<br>◯ > 200 copias/mL<br>(Última determinación realizada durante el último año)
TAR actual	◯ Yes ◯ No
Régimen TAR actual	◯ 2 nucleósidos + 1 no-nucleósido<br>◯ 2 nucleósidos + 1 IP<br>◯ 2 nucleósidos + 1 Inh Int<br>◯ Biterapia con DTG + 3TC o RPV<br>◯ Biterapia con IP + 3TC<br>◯ Biterapia otras combinaciones<br>◯ Monoterapia con IP<br>◯ Otras pautas
Especificar:	__________________________________

02/11/2023 4:15pm projectredcap.org REDCap®

Categoría de este régimen TAR:	◯ Pauta de inicio<br>◯ Cambio sin fracaso ni toxicidad (Switch)<br>◯ Cambio por fracaso<br>◯ Cambio por toxicidad<br>◯ Estudio / ensayo clínico
Serología positiva para VHC	◯ Si<br>◯ No<br>◯ Desconocido
ARN VHC positivo actualmente<br><br>Todo paciente en tratamiento anti-VHC actual o pendiente de resultado de RVS12 se considera infección activa (ARN-VHC+)	◯ Si<br>◯ No<br>◯ Desconocido
¿Cuándo ha ocurrido la infección?	◯ En el último año<br>◯ Hace más de un año<br>◯ Desconocido
Genotipo y subtipo de VHC	◯ 1a<br>◯ 1b<br>◯ 2<br>◯ 3<br>◯ 4<br>◯ 5<br>◯ 6<br>◯ Mixto<br>◯ Desconocido
Seleccionar lo que aplique:	◯ Aclaramiento espontáneo<br>◯ Tras tratamiento anti-VHC
¿Está recibiendo tratamiento VHC o está pendiente de confirmar RVS 12?	◯ Yes ◯ No
Tipo de tratamiento:	◯ SOF + LDV ± RBV<br>◯ SOF + VEL ± RBV<br>◯ SOF + VEL + VOX ± RBV<br>◯ GLE + PIB ± RBV<br>◯ EBR/GZR ± RBV<br>◯ OBV/PTV/r + DSV ± RBV (3D)<br>◯ OBV/PTV/r ± RBV (2D)<br>◯ Otros
Especificar:	______________________________
¿Porqué no recibe tratamiento anti-VHC este paciente?	◯ Negativa del paciente<br>◯ Pérdida de seguimiento<br>◯ Criterio médico<br>◯ Previsión de iniciar tratamiento o retratamiento a corto plazo<br>◯ Otras
Especifique:	______________________________

¿Ha recibido con anterioridad el tratamiento?	◯ Yes ◯ No
Tipo de tratamiento (el último recibido):	◯ IFN +/- RBV sin AADs<br>◯ IFN + RBV + AADs<br>◯ AADs (libre de IFN)<br>◯ Otros
Especificar:	__________________________________
Desenlace del último tratamiento anti-VHC	◯ Respuesta viral sostenida<br>◯ Fracaso virológico<br>◯ Retirada / abandono por toxicidad
HBsAG positivo	◯ Si<br>◯ No<br>◯ Desconocido
Anti VHD positivo	◯ Si<br>◯ No<br>◯ Desconocido
ARN VHD (última determinación)	◯ Positivo<br>◯ Negativo<br>◯ No realizado / No disponible
Fármacos anti-VHB utilizados (marcar todos los que corresponda)	☐ 3TC<br>☐ FTC<br>☐ TDF<br>☐ Entecavir<br>☐ Peg IFN<br>☐ TAF<br>☐ Ninguno
ADN-VHB en los últimos 12 meses	◯ Yes ◯ No
Marcar lo que corresponda:	◯ < 80 UI/mL ◯ 80-2000 UI/mL<br>◯ >2000 UI/mL
Diagnóstico previo / actual de cirrosis	◯ Yes ◯ No
Método de diagnóstico de la cirrosis	☐ Biopsia hepática (F4 METAVIR o similar)<br>☐ Fibroscan (> 12,5 kPa)<br>☐ Criterio clínico / biológico
Causas potencialmente implicadas en la cirrosis de este paciente	☐ Infección por virus C<br>☐ Infección por virus B<br>☐ Alcoholismo<br>☐ Esteatohepatitis<br>☐ Otras
Cribado hepatocarcinoma con ECO/TC/RM:	◯ Realizado en últimos 6 meses<br>◯ Realizado entre últimos 6 - 12 meses<br>◯ No realizado en los últimos 12 meses
Descompensación hepática previa/actual	◯ Yes ◯ No

Hepatocarcinoma previo/actual	◯ Yes ◯ No
Ascitis actual	◯ Sin ascitis<br>◯ Moderada (controlada con diuréticos)<br>◯ Severa
Encefalopatía actual	◯ Sin encefalopatía<br>◯ Grado I a II<br>◯ Grado III a IV
Trasplante hepático previo o actual	◯ Yes ◯ No
Albúmina actual g/dL	__________________________________<br>(Última determinación realizada durante el último año)
Bilirrubina actual mg/dL	__________________________________<br>(Última determinación realizada durante el último año)
Última cifra de INR	__________________________________<br>(Última determinación realizada durante el último año)
Creatinina actual mg/dL	__________________________________<br>(Última determinación realizada durante el último año)
ALT actual U/L	__________________________________<br>(Última determinación realizada durante el último año)
AST actual U/L	__________________________________<br>(Última determinación realizada durante el último año)
Cifra de plaquetas x 103/uL actual	__________________________________<br>(Última determinación realizada durante el último año)

FIBROSCAN

Fibroscan ◯ Yes ◯ No

Fecha del último estudio:
____

Valor del último estudio:
____

Appendix 2. Participating centers in the nine prevalence studies of HCV in Spain conducted between 2002 and 2023

#	Center
1	Hospital General Universitario Gregorio Marañón, Madrid
2	Hospital Universitario La Paz, Madrid
3	Hospital Universitario Ramón y Cajal, Madrid
4	Hospital San Pau i Santa Creu, Barcelona
5	Hospital Universitario 12 de Octubre, Madrid
6	Hospital Clínico San Carlos, Madrid
7	Hospital Universitario Infanta, Madrid
8	Hospital Universitario Vall d´Hebrón, Barcelona
9	Hospital Universitario Virgen de la Victoria, Málaga
10	Hospital Universitario Álvaro Cunqueiro, Vigo
11	Hospital Donostia, San Sebastián
12	Hospital Miguel Servet, Zaragoza
13	Hospital de la Princesa, Madrid
14	Hospital Clínico de Valencia, Valencia
15	Hospital Doctor Peset, Valencia
16	Hospital Reina Sofía, Córdoba
17	Hospital General de Alicante, Alicante
18	Hospital Universitario Marqués de Valdecilla, Santander
19	Hospital Universitario Basurto, Bilbao
20	Hospital Virgen de las Nieves, Granada
21	Hospital La Fe, Valencia
22	Hospital Son Llátzer, Palma de Mallorca
23	Hospital de Cabueñes, Gijón
24	Hospital Universitario Príncipe de Asturias, Alcalá de Henares
25	Hospital Universitario de Getafe, Getafe
26	Hospital Fundación de Alcorcón, Alcorcón
27	Hospital Reina Sofía, Murcia
28	Hospital Joan XXIII, Tarragona
29	Hospital Universitario San Pedro, Logroño
30	Hospital General de Albacete, Albacete
31	Hospital de Mataró, Mataró
32	Hospital Universitario de Torrejón, Torrejón de Ardoz
33	Hospital Virgen de la Cinta, Tortosa
34	Hospital Rafael Méndez, Lorca
35	Hospital Virgen de la Concha, Zamora
36	Hospital Universitario Infanta Elena, Valdemoro
37	Hospital San Eloy, Barakaldo
38	Hospital de Sierrallana, Torrelavega
39	Hospital Virgen de la Luz, Cuenca
40	Centro Sanitario Sandoval, Madrid
41	Fundació Hospital Sant Jaume, Olot.
42	Hospital Universitario Arnau de Vilanova, Lleida
43	Hospital Universitario Santa María, Lleida

Appendix 3. GeSIDA 8514 Study Group

Hospital General Universitario Gregorio Marañón: C Fanciulli, L Pérez-Latorre, P Miralles, JC López, F Parras, B Padilla, T Aldámiz, C Díez, F Tejerina, J Berenguer.
Hospital Universitario La Paz: MM Arcos, C Busca, A Delgado, V Hontañón, R de Miguel, R Micán, JR Arribas, JI Bernardino, ML Martín-Carbonero, R Montejano, ML Montes, L Ramos, E Valencia, J González-García.
Hospital Universitario Ramón y Cajal: MJ Vivancos-Gallego, S Moreno, A Moreno, JL Casado, MJ Pérez-Elías, C Quereda.
Hospital Santa Creu i Sant Pau: J Muñoz, L Millán, M Gutiérrez, G Mateo, JM Guardiola, P Domingo.
Hospital Universitario 12 de Octubre: A Hernando, L Domínguez, O Bisbal, R Rubio, F Pulido.
Hospital Clínico San Carlos: J Barrado, J Vergas, MJ Téllez, V Estrada.
Hospital Universitario Infanta Leonor: P Ryan.
Hospital Universitari Vall d´Hebrón: J Navarro, A Torrella, N Ramos, B Planas, M Sanchiz.
Hospital Virgen de la Victoria: J Ruiz, E Nuño, M Márquez, J Santos, R Palacios, C Gómez, M López.
Hospital Universitario Alvaro Cunqueiro: S Castro, M Crespo, L Morano.
Hospital Universitario de Donostia: M Ibarguren, MP Carmona, F Rodríguez-Arrondo, MA Goenaga, H Azkune, MA Von Wichmann, JA Iribarren.
Hospital Universitario Miguel Servet: P Arazo, J Moreno, R Martínez, R Caballero, A Caudevilla.
Hospital Clínico Universitario de Valencia: R Ferrando-Vilalta, A Ferrer, MJ Galindo.
Hospital Universitario de la Princesa: L García-Fraile, S Otero, A Bautista, I de los Santos.
Hospital Universitario Doctor Peset: I López-Cruz, J Carmena, A Artero.
Hospital Universitario Reina Sofía: A Camacho, IM Machuca, A Rivero-Juárez, I Reyes, I Ruiz, A Rivero-Román.
Hospital General Universitario Dr. Balmis: L Giner, S Reus, M Carreres, E Merino, V Boix, D Torrús, I Portilla, M Pampliega, M Díez, I Egea, J Portilla.
Hospital Universitario Marqués de Valdecilla: F Arnaiz, C Armiñanzas, S Echevarría, M Gutiérrez-Cuadra, MC Fariñas.
Hospital Universitario Virgen de las Nieves: C Hidalgo, C García.
Hospital Universitario y Politécnico La Fe: M Montero, M Tasias, S Cuellar, E Calabuig, M Blanes, J Fernández, I Castro, J López-Aldeguer, M Salavert.
Hospital Universitario de Basurto: OL Ferrero, S Ibarra, I López, M de la Peña, Z Zubero, J Baraia, J Muñoz.
Hospital Universitario Son Llátzer: A Villoslada, C Cifuentes.
Hospital Universitario de Cabueñes: JF Soler-González, M Campoamor, G López, B de la Fuente, V Arenas.
Hospital Universitario Príncipe de Asturias: C Hernández, M Novella, J Sanz.
Hospital Universitario de Getafe: S Rodríguez, G Gaspar.
Hospital Fundación de Alcorcón: M Velasco, L Moreno, R Hervás, JE Losa.
Hospital Universitario Reina Sofía de Murcia: E García-Villalba, C Tomás, S Valero, JM Gómez, R Martínez, MD Hernández, MI Martínez, A Alcaráz, A Muñoz, E Bernal.
Hospital Universitari de Tarragona Joan XXIII: F Vidal, M Vargas, A Castellano, J Peraire, C Viladés, S Veloso, B Villar.
Hospital Universitario San Pedro: L Pérez-Martínez, JA Oteo.
Complejo Hospitalario Universitario de Albacete: F Mateos, E Martínez-Alfaro.
Hospital de Mataró: L Arbonés, L Force, P Barrufet.
Hospital Verge de la Cinta: AJ Orti, E Chamarro, C Escrig.
Hospital Universitario de Torrejón: D Corps, A Gimeno, C Montero, S Arponen.
Hospital Virgen de la Concha: C Martín, A Chocarro.
Hospital Rafael Méndez: G Alonso, AI Peláez, C Toledo, G Lara, I Fernández, MC Esteban.
Hospital Universitario Infanta Elena: M Clavero, V Víctor.
Hospital San Eloy-OSI: R Silvariño, J Ugalde.
Hospital de Sierrallana: R Teira.
Hospital Virgen de la Luz: O Belinchón, P Geijo.
Hospital Universitario Clínico San Cecilio: D Vinuesa, L Muñoz, J Hernández-Quero.
Centro Sanitario Sandoval: C Rodríguez, T Puerta, M Raposo, M Vera, J Del Romero.
Hospital d'Olot i Comarcal de la Garrotxa: J Bisbe.
Hospitales Universitarios Arnau de Vilanova y Santa María: T Puig.
Instituto de Salud Carlos III: I Jarrín.
Fundación SEIMC-GESIDA: M De Miguel, H Esteban.
